# Multi-Analyte MS Based Investigation in Relation to the Illicit Treatment of Fish Products with Hydrogen Peroxide

**DOI:** 10.3390/toxics8010002

**Published:** 2020-01-08

**Authors:** Federica Dal Bello, Riccardo Aigotti, Michael Zorzi, Valerio Giaccone, Claudio Medana

**Affiliations:** 1 Molecular Biotechnology and Health Sciences Dept. Università degli Studi di Torino, Via Pietro Giuria 5, 10125 Torino, Italy; riccardo.aigotti@unito.it (R.A.); michael.zorzi@unito.it (M.Z.); claudio.medana@unito.it (C.M.); 2 Department of Animal Medicine, Productions and Health, Università degli Studi di Padova, Viale dell’Università 16, 35020 Legnaro, PD, Italy; valerio.giaccone@unipd.it

**Keywords:** mass spectrometry methods, fishery product, hydrogen peroxide, illicit treatment

## Abstract

Fishery products are perishable due to the action of many enzymes, both endogenous and exogenous. The latter are produced by bacteria that may contaminate the products. When fishes age, there is a massive bacteria growth that causes the appearance of *off-flavor*. In order to obtain “false” freshness of fishery products, an illicit treatment with hydrogen peroxide is reported to be used. Residues of hydrogen peroxide in food may be of toxicology concern. We developed two mass spectrometry based methodologies to identify and quantify molecules related to the treatment of fishes with hydrogen peroxide. With ultra-high-performance liquid chromatography–mass spectrometry (UHPLC-MS) we evaluated the concentration of trimethylamine-N-oxide (TMAO), trimethylamine (TMA), dimethylamine (DMA), and cadaverine (CAD) in fish products. After evaluating LOQ, we measured and validated the lower limits of quantification (LLOQs as first levels of calibration curves) values of 50 (TMAO), 70 (TMA), 45 (DMA), and 40 (CAD) ng/mL. A high ratio between TMAO and TMA species indicated the freshness of the food. With a GC-MS method we confirmed the illicit treatment measuring the levels of H_2_O_2_ after an analytical reaction with anisole to give 2-hydroxyanisole as a marker. This latter product was detected in the headspace of the homogenized sample with simplification of the work-up. A LLOQ of 50 ng/mL was checked and validated. When fish products were whitened and refreshed with hydrogen peroxide, the detected amount of the product 2-hydroxyanisole could be very important, (larger than 100 mg/kg). The developed analytical methods were suitable to detect the illicit management of fishery products with hydrogen peroxide; they resulted as sensitive, selective, and robust.

## 1. Introduction

Fishery products are defined by Regulation (EC) N 853/2004 of the European Parliament and the Council as “all seawater or freshwater animals (except for live bivalve mollusks, live echinoderms, live tunicates and live marine gastropods, and all mammals, reptiles and frogs) whether wild or farmed and including all edible forms, parts and products of such animals” [1]. The description includes all fishes (Osteichthyes, bony fishes, and Chondrichthyes, cartilaginous fishes), shellfish, and clams.

The fishery products are classified in four commercial categories: Fresh (no manipulation), prepared (any operations that affect the anatomical wholeness of the animal), frozen, and processed (any operation that transform the product, such as smoking, marinating, salting). The freshness of a fish product is the most important commercial quality factor for the consumer, because the safety is an essential prerequisite without which food cannot be placed on the market or further transformed. The Regulation (EC) N 2406/96 of the European Parliament and the Council defines four categories for fresh fish products (extra, a, b, and c); fishery products grouped in the last class must be judged as not suitable for human consumption and must be removed from the market [2]. 

Fishery products are perishable due to the action of many enzymes, both endogenous, located in the fish muscles, and exogenous, produced by intrinsic bacteria that are present and can contaminate in the products [3]. Consequently, the exponential growth of bacteria that triggers oxidative chemical reactions causes fading and opacification of the product, with appearance of *off-flavor*, that is an unpleased flavor caused by chemical lipid oxidation or non-protein nitrogen (NPN) degradation [3,4]. NPN in fishery products are distinguished in two structural categories: Volatile and nonvolatile compounds. Nonvolatile NPN compounds are mostly represented by heterocyclic metabolites while volatile ones own low molecular weight and are represented in fishery products mainly by ammonia, trimethylamine (TMA, C_3_H_9_N), and dimethylamine (DMA, C_2_H_7_N). TMA is an endogenous compound abundant in fishery products: It is a post-mortem product, deriving from trimethylamine-N-oxide (TMAO, C_3_H_9_NO) by an enzymatic activity [5,6,7,8].

TMAO is an amine oxide, less volatile, and less basic with respect to TMA, due to its oxidation. Under enzymatic activity, TMAO could generate several chemical compounds: DMA and formaldehyde (FA) from endogenous (aquatic environment) muscle bacteria (*Pseudomonas* and *Alteromonas*) activity, and TMA from exogenous (which accumulates in fish products after capture and is typical of the terrestrial environment) bacteria (*Salmonella*, *Vibrio*) activity [9]. Only a limited population of bacteria can cause deterioration of fishery products: They are called specific spoiling micro-organism (SSO) and are mostly Gram-negative micro-organisms. After fishing, SSO can contaminate fishery products on the surface and grow even at low temperatures [10,11,12,13]. DMA, FA, and TMA are products of enzymatic degradation by intrinsic bacteria together with biogenic amines (histamine, tyramine, phenylethylamine, putrescine, and cadaverine), thiols and hydrogen sulfide (H_2_S). Moreover, in mollusks such as squid, the SSO enzymes can produce hypoxanthine and acetic acids salts, that contribute to the appearance of *off-flavor* [14,15].

The organo-nitrogen compounds derived from degradation of NPN and protein are quantifiable as a total volatile basic nitrogen TVB-N; for fresh fish products the TVB-N amount should be minor of 10 mg/kg, for aged ones the quantity ranges normally between 300–350 mg/kg (Regulation (EC) N 853/2004) [16,17,18]. Due to the unhealthiness, the *off-flavor* appearance, and color changes, aged fishery products must be retired from the market. However, some illicit treatments on these products might simulate a “false” freshness and one of these treatments is the use of hydrogen peroxide (H_2_O_2_). The illicit treatment with 0.5%–0.8% hydrogen peroxide water solutions is known and was reported in the literature [19]. Residues of hydrogen peroxide in food may be of toxicology concern.

Hydrogen peroxide is both an oxidant in aqueous solution with acidic pH and a reductant in alkaline water solution. When it is used as an illicit treatment for fishery products, the oxidizing properties are exploited. H_2_O_2_ can indeed convert TMA, a degradation product, to TMAO, the amine oxide naturally present in living fishes. TMAO is odorless and it has oxidizing properties giving to the fishery substrate high redox potential. This great redox potential is typical of muscle tissue of fresh fishery products, and decreases rapidly when TMAO is reduced to TMA by enzymatic activity [20,21,22].

If the TMAO amount is increased by H_2_O_2_ treatment, the proteins are stabilized [23]. For example, the amount of mucins, the main glycoproteins of mucus, is reduced on the fish skin after H_2_O_2_ treatment because of chemical degradation. The decrease in mucins concentration reduces viscosity and slows down the appearance of *off-flavor* [24].

Finally, the illicit treatment with H_2_O_2_ can cause a whitening and “re-freshing” effect on fishery products due to its oxidative properties (peroxidation of double bonds present in chromophores) [25,26].

The aim of the present study was the development of mass spectrometry (MS) based methods to evaluate the concentration of different compounds related to the illicit treatment of fish food based on the use of hydrogen peroxide (whitening and “re-freshing”). For this purpose, both liquid chromatography and gas chromatography hyphenated mass spectrometers were used. Mass spectrometry is recognized as one of the powerful and sensitive analytical techniques to identify, characterize, and quantify small molecules, such as amines and ethers. The use of this kind of MS based techniques is worthwhile in food analysis requiring complementary approaches to the detection of chemicals with different physical–chemical properties such as in the present work [27,28,29].

Two analytical methods were developed: The first one was a direct LC-MS/MS method for the determination of various amines and trimethylamine-oxide (TMAO); the second one was an indirect SPME-GC-MS method for the determination of residues of H_2_O_2_ on different fishery products matrices, by the hydroxylation reaction of anisole to 2-hydroxyanisole (guaiacol).

Another aim of this work was the application of the developed methods to investigate about H_2_O_2_ fish products treatment. The consequent alteration of the concentration ratio of TMAO and trimethylamine (TMA) which is a known fish product freshness parameter was evaluated.

## 2. Materials and Methods

All solvents and analytical standards of dimethylamine (DMA), trimethylamine (TMA), trimethylamine-N-oxide (TMAO), cadaverine (CAD), hydrogen peroxide solution, anisole, guaiacol, and p-xylene-d10 were purchased from Sigma Merck (Merck, Milan, MI, Italy). High-performance liquid chromatography (HPLC)-grade water was obtained from a MilliQ Academic water purification system (Millipore, Milan, Italy). Before use, solvents were filtered through a 0.45 µm filter and degassed for 10 min in an ultrasonic bath.

Fishery products samples were: Atlantic bonito (*Sarda sarda*) mackerel-like fish of the family Scombridae; European squid (*Loligo vulgaris*) belonging to the family Loliginidae. Atlantic bonito and European squid samples were purchased in a local market or caught fresh in Tyrrhenian Sea and brought in the laboratory no later than 5 h after fishing, in ice.

### 2.1. UHPLC-Tandem Mass Analysis of NPN

A Nexera (Shimadzu, Milan, MI, Italy) UHPLC (Ultra-high-performance liquid chromatography) coupled through an ESI source with a QTRAP-5500 tandem mass analyzer (Sciex, Milan, MI, Italy) was used to quantify the NPN in fishery samples.

The chromatographic separation was achieved using a RP C18 column (Kinetex EVO, 5 µm, 150 × 2.1 mm, Phenomenex, Castel Maggiore, BO, Italy) and heptafluoro-butanoic acid 10 mM in water (eluent A) and in methanol (eluent B). The gradient run started with 1% B, increased to 35% B in 8 min, up to 100% in 3 min, followed by reconditioning time. Flow rate and injection volume were 200 µL min^−1^ and 5 µL, respectively.

The triple quadrupole was used in a MRM positive ion mode with the following source parameters: Curtain gas (arbitrary unit, arb), 25; spray voltage (V), 5500; gas1 (arb), 35; gas2 (arb), 40; capillary temperature (°C), 400. Nitrogen was used as curtain gas, gas 1, and gas 2. The MRM transitions, potentials, and collision energies were listed in Table 1. 

The developed analytical method was validated evaluating selectivity, linearity, accuracy and precision, and lower limit of quantitation following the FDA guidelines [30].

### 2.2. Gas Chromatography–Mass Spectrometry Analysis of H_2_O_2_ Residue

For the GC-MS analysis we used a Varian Saturn 3900 (Agilent, Milan, MI, Italy) system, equipped with a 1177 injector. The separation column was a Zebron ZB-624 30 m, i.d. 0.25 mm (Phenomenex, Castel Maggiore, BO, Italy) applying a temperature gradient from 40 to 240 °C in 16 min. Injector temperature was 240 °C; split mode was employed and helium gas flow was 1.2 mL min^−1^.

The mass spectrometry was a Varian Saturn 2100 T ion trap analyzer (Agilent, Milan, MI, Italy) equipped with an EI (electron ionization) source. The full mass acquisition range was from 40 to 500 *m/z*.

The developed methodology was based on the paper of Tanaka et al. [31] calibrating the modified procedure in order to measure H_2_O_2_ concentration in the range 0.05 to 1.00 µg/mL. 

### 2.3. Sample Preparation for UHPLC/GC-MS Analysis of NPN

Samples of fish products muscles were weighted, minced, and extracted with pH 2.5, 0.1 M phosphate buffer: 40 mL of buffer were used for 8 g of fish sample; the suspensions were then centrifuged (2300 *g* for 10 min) and filtered (0.45 µm).

Some samples were treated with hydrogen peroxide to simulate the illicit treatment: After a total immersion of 8 g of fish products in H_2_O_2_ solution (0.8%) for 2 min, the liquid was removed and samples were rinsed with fresh water. Then, treated fishery products samples were extracted as just described.

For UHPLC-MS, 1 mL of extracted solution was diluted using the starting eluent mixture, placed in a vial, and analyzed in a MRM mode using the triple quadrupole in a positive ion mode.

For GC-MS, 2 mL of extracted solution were placed in a vial for headspace solid-phase microextraction (HS SPME) and added with 100 µL of potassium ferricyanide (K_8_Fe(CN)_6_) as catalyzer and 2 µL of anisole. The solution was heated at 60 °C in an oil bath for 1 h; the fiber for head space analysis was exposed for 30 min. A Supelco 75 µm Carboxen™-PDMS (polydimethylsiloxane) (Merck, Milan, MI, Italy) fiber was used. The extraction recovery of anisole was checked to be 80% by the use of p-xylene-d10 as an internal standard for 30 min fiber exposition.

## 3. Results

With the developed MS based methodologies we were able to quantify the amount of the amines dimethylamine (DMA), trimethylamine (TMA), trimethylamine-N-oxide (TMAO), and cadaverine (CAD) with the UHPLC-tandem mass analysis. The concentration of H_2_O_2_ residues was measured with the use of a HS SPME-GC-MS method.

### 3.1. Results of UHPLC-Tandem Mass Analysis of NPN

**Figure 1:**
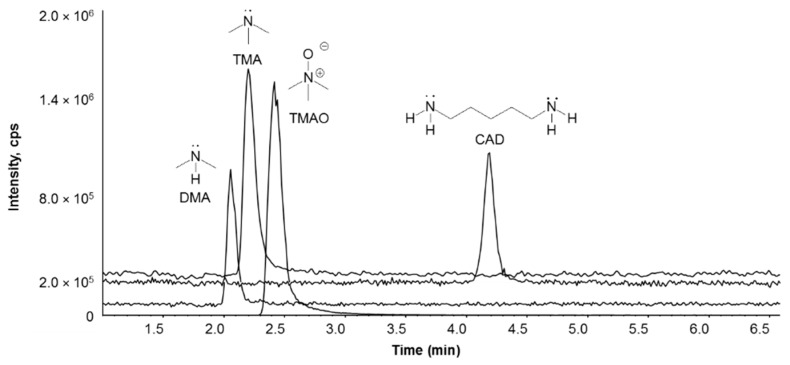
Chromatographic separation of dimethylamine (DMA, R_t_ 2.2 min), trimethylamine (TMA, R_t_ 2.25 min), trimethylamine-N-oxide (TMAO, R_t_ 2.5 min), and cadaverine (CAD R_t_ 4.3 min).

The chromatographic separation of the analyzed amines is shown in Figure 1. The ion pairing effect of the heptafluoro-butanoic acid present in the mobile phase allowed a valuable retention to obtain a satisfying separation of the analytes.

In order to quantify the amines in fresh fishery products samples or in fishery products subjected to an illicit treatment with H_2_O_2_, three calibration curves were prepared: (I) In pure pH 2.5, 0.1 M phosphate buffer, (II) In the extraction buffer fresh samples of Atlantic bonito, and (III) In the extraction buffer of fresh samples of European squid.

**Figure 2:**
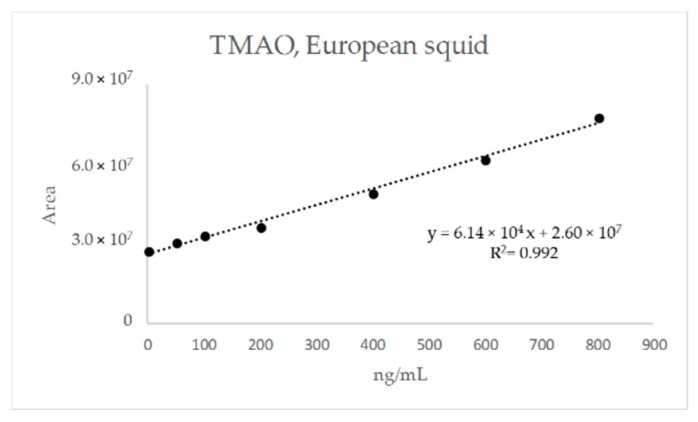
A standard addition curve of TMAO in a real sample of fresh European squid. The basal concentration of TMAO was 1150 ng/mL.

Each matrix material was weighted and extracted as previously described; since the matrix has a basal amount of amines, it was mandatory to prepare a matrix-blank without the addition of analyte (DMA, TMA, TMAO, and CAD) standards. Once obtained, the matrix-blank and the calibration curves were obtained by adding increasing amounts of amines as follows: 50, 100, 200, 400, 600, 800 ng/mL. A standard addition curve of TMAO in a real sample of European squid is shown in Figure 2. The curve had a positive intercept value because of the basal amounts of the analyte in fish.

A full validation of the UHPLC-MS method for amines determination was performed. We followed the Food and Drug Administration (FDA) guidelines to evaluate the protocol [30] and used our previous work as an example for validation parameter definition [32]. Validation parameters were listed in Table 2 for all the UHPLC-MS analytes. LOD and LOQ were evaluated on the standard calibration curve by the signal to noise values of 3 and 10, respectively. LLOQ is the lower limit of quantification determined on the basis of a simple LOQ. To obtain LLOQs, LOQ standard solutions were prepared, used as the first level of each calibration curve, and validated by comparison of blank solutions. Validation parameters definition is given in the following paragraph.

To test the possible interferences at the analyte of interest’s known retention time and *m/z*, a standard solution that had every analyte at a known concentration except for the one of interest, was analyzed. The selectivity % (Sel% = (Area at analyte retention time/Average area analyte in LLOQ) × 100) had to be ≤30%. The statistical parameter related to the linearity of calibration curve is the percentage difference (Diff% = (slope − average slope)/ average slope) × 100). Diff% had to be ≤25%. Inaccuracy of lower limit of quantitation (LLOQ) had to be ≤20% and relative standard deviation % of accuracy of LLOQ ≤15%. Finally, to test recovery, matrices were spiked with a combined standard solution of amines at known concentration and processed as described in the Sample preparation section. The recovery (Rec = (Area at analyte retention time in spiked matrix/Average analyte area in spiked water solution) × 100) had to be between 85% and 120%. The validation parameters were respected in all cases.

The UHPLC-tandem mass method was then applied to real fishery product samples of Atlantic bonito (*Sarda sarda)* and European squid (*Loligo vulgaris*): A) Freshly caught; B) freshly purchased in a local market; C) aged (four days at room temperature); and D) H_2_O_2_ treated (as described before).

The obtained results are summarized in Table 3. In freshly caught and freshly purchased samples the measured amount of amine was similar; in Table 3 the quantity of amines in freshly caught samples only was reported. High levels of TMAO were found in freshly caught Atlantic bonito (1700 ± 238 mg/kg) and European squid (1200 ± 336 mg/kg) samples. Conversely, in these samples the TMA amount was low, 170 ± 24 and 210 ± 29 mg/kg, respectively.

When fishery products initiated to degrade due to temperature (4 h at room temperature) or bacterial activities, the TMAO and TMA amounts reversed. Finally, when hydrogen peroxide was used as an illicit treatment as whitening and refreshing agents, exploiting its oxidant properties, the balance was shifted again towards a higher level of TMAO. In *Sarda sarda* the values after H_2_O_2_ treatment were TMAO 410 ± 57, TMA 720 ± 180 mg/kg; and in *Loligo vulgaris* were TMAO 850 ± 212, TMA 200 ± 28 mg/kg.

### 3.2. Results of GC-MS Analysis of H_2_O_2_ Residues

**Scheme 1:**
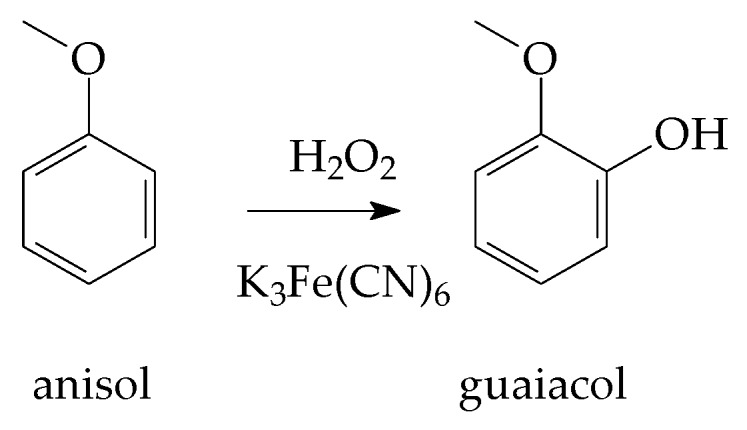
Oxidation reaction with hydroxyl peroxide of anisole to guaiacol (2-hydroxyanisole) catalyzed by potassium ferricyanide.

To confirm the illicit management of fishery products with hydrogen peroxide, we implemented a previously published GC-ECD assay based on peroxide detection by indirect oxidation of anisole to guaiacol (2-hydroxyanisole) (Scheme 1) [31].

We developed a headspace solid-phase microextraction (HS SPME) GC-MS methodology to improve sensitivity, easiness of operation, and reliability.

**Figure 3:**
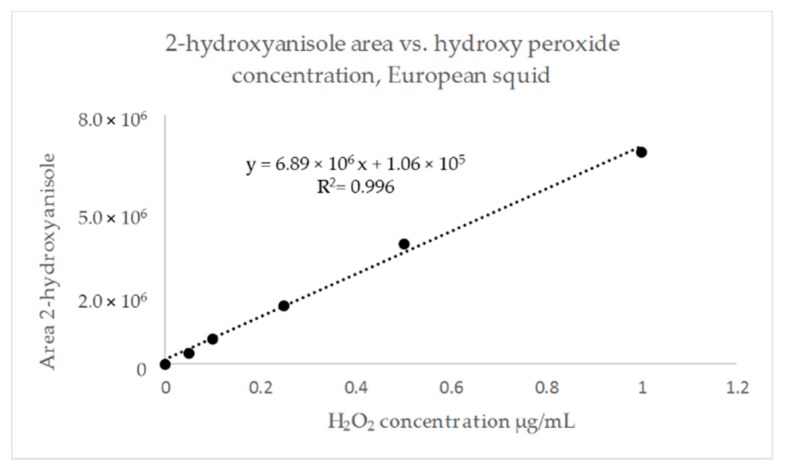
A standard addition curve of H_2_O_2_ in a fresh sample of European squid.

To quantify the residues of H_2_O_2_ we prepared a calibration curve with the standard addition method using fresh fish product samples of European squid and Atlantic bonito as matrices. The extracted solution of fresh fishery products was added with aliquots of hydrogen peroxide to give amounts of 0.0, 0.05, 0.1, 0.25, 0.5, and 1.0 µg/mL of H_2_O_2_. Then, 100 µL of potassium ferricyanide (K_8_Fe(CN)_6_) and 2 µL of anisole were added to the obtained solutions before fiber exposing for head space analysis. We measured the 2-hydroxyanisole peak area which was related to the H_2_O_2_ addition. Figure 3 shows a standard addition calibration curve of H_2_O_2_ in European squid samples. The obtained LLOQ was 50 ng/mL. When the concentration of hydrogen peroxide was zero, no 2-hydroxyanisole was detected.

We then applied the developed HS SPME-GCMS method to five real fishery products samples, in particular squid which were the subject of illicit treatment with hydrogen peroxide due to its properties of whitening agent.

QC and real samples were prepared as described in the Sample Preparation section: The amount of H_2_O_2_ added for the redox reaction of anisole to guaiacol was set at 0.5 µg/mL. In these conditions we tested a blank solution without fishery products (no *Sarda sarda* or *Loligo vulgaris*), fresh (blank matrix), fresh and treated in controlled conditions (QC) and illicit treated fishery products samples of Atlantic bonito and European squid. All of the five samples coming from legal controls showed H_2_O_2_ values higher than 100 ppm. Table 4 shows the results. The peak area of 2-hydroxyanisole in the buffer without matrices confirmed the added amount of H_2_O_2_ of 0.5 ± 0.07 ppb. In the presence of matrices, the peak area decreased due to the matrix effect. In the case of fresh fish product samples, this effect was quantifiable in a loss of 12% of H_2_O_2_ amount (0.44 ± 0.11 µg/mL for Atlantic bonito and 0.43 ± 0.10 µg/mL for European squid). In fresh samples treated with hydrogen peroxide in controlled conditions (complete immersion of samples in a 0.8% H_2_O_2_ solution followed by rinsing with fresh water in laboratory) the amount of H_2_O_2_ was much higher than 1 ppm, the highest calibration curve point. However, extrapolating the results, it seemed larger than 100 ppm. The same was for the samples, especially squid, illegally treated with hydrogen peroxide.

## 4. Discussion

The developed mass spectrometry based analytical methods were adequately selective to quantify the amount of peculiar molecules related to the illicit treatment of fishery products with hydrogen peroxide. In particular, to quantify trimethylamine, trimethylamine-N-oxide, dimethylamine, and 2-hydroxyanisole.

Many other analytical methods were proposed to measure the analytes [33,34,35,36,37,38,39,40,41]. The following paragraph gives a summary about was presented.

Bilgin’s and Chung‘s research groups performed a quantitation of some amines in a large number of fish samples using HPLC coupled to a photodiode array detector [33] or other detectors such as chemiluminescent nitrogen, SPME-GC-MS, and spectrophotometric ones [34]. The first study regarded the determination of histamine, cadaverine, and tyramine after derivatization with dansyl chloride in 63 fish samples. The declared limit of quantitation was in the range between 0.010 and 0.100 µg/mL. Chung et al. determined TMAO, TMA, DMA, and FA in 266 fish samples. They were used respectively for TMAO, HPLC coupled to a chemiluminescent detector; for the TMA and DMA SPME-GC-MS method with a carboxen/divinylbenzen/polydimethylsiloxane fiber; and LC-visible analyzer for FA after derivatization with 2,4-dinitrophenylhydrazine. The stated LOQ were 25, 10, 10, 5 mg/kg for TMAO-DMA-TMA-FA, respectively [34]. Moreover, Soncin [40] and Chan et al. [35] in their studies used as analytical methodologies the headspace solid-phase extraction coupled with GC-MS. The first performed an identification and quantitation of markers of spoilage in fish and found as indicators four molecules. They defined the technique suitable for analyzing the volatile compounds [40]. Chan’s study monitored the concentration of DMA and TMA in fish and the achieved limit of detection was 100 ppb [35].

Heude in his paper published on Food Analytical Methods in 2015 used a ^1^H high resolution magic angle spinning NMR spectroscopy to determine the *K*-value [36] and trimethylamine nitrogen content as parameters of freshness and quality of fish products. They studied four species of fish and highlighted as great advantages the possibility to not process fish samples, no extraction was required for NMR analysis [36]. Feng Li et al. in their research used ion chromatography coupled to an unsuppressed conductivity detector to measure the amount of DMA, TMA, and TMAO in aquatic products. They obtained a good lower limit of detection (60, 80, 100 ng/mL for DMA-TMA-TMAO) and quantified the analytes in three real samples [38].

Finally, the remaining methods developed [37,39,41] were based on mass spectrometry techniques in order to determine the amines of interest. Le’s group using a C18-PFP column and triple quadrupole quantify TMAO in human plasma samples as a potential indicator of cardiovascular health. The determined LOD and LOQ were 1 and 6 ng/mL [37]. Romero-Gonzáles et al. measured cadaverine and TMA with UHPLC-MS with a LLOQ of 25 and 60 ppb, respectively [39]. Finally, using a hydrophilic interaction liquid chromatography coupled to mass spectrometry Wu et al. quantified in fish meals five amines with satisfactory LOD and LOQ [41].

The present method is based on ultra-high liquid chromatography coupled to mass spectrometry and demonstrates to be sufficiently sensitive to quantify the four studied amines in fishery products in 5 min only. As previously indicated, the lower limits of quantification in matrices for DMA-TMA-TMAO and CAD were 45, 70, 50, and 40 ng/mL, respectively. The selectivity of the method was remarkable in spite of the low mass to the charge value of molecular protonated ions of the analytes. This region of low *m/z* ratio ([M+H]^+^ 46, 60, 76, and 103 for DMA-TMA-TMAO, and CAD) is normally affected by a high background noise, but using tandem mass spectrometry the signal to noise ratio was reasonably good.

To highlight the illicit treatment of fishery products with hydrogen peroxide, we focused our attention on the ratio between TMAO and TMA. As shown in Table 3, subsequently to the prohibited washing with H_2_O_2_, the ratio between the measured compounds was shifted again towards a higher concentration of TMAO, one of the endogenous molecular marker of freshness. This ratio was not completely reversed because hydrogen peroxide acted only on the skin of the fishery products, besides the alkylamine is present in higher concentration in muscles. However, the treatment could make the product look younger and fresher.

As explained in the results section, the GC-MS method was an upgrade of the Tanaka et al. GC-ECD methodology for the quantitation of hydrogen peroxide in Chinese foods. The quantitation is possible thanks to the indirect measurement of 2-hydroxyanisole generated by a potassium ferricyanide catalyzed redox reaction of anisole with H_2_O_2_ [31]. In that paper, Tanaka et al. performed a deep investigation about reaction environment (pH range), catalyzer, and hydrogen peroxide concentrations in order to obtain the highest possible yield of redox reaction. For ECD quantitation, pentafluoro-benzoyl chloride was used as a derivatizing agent prior to analysis of the product of the reaction, 2-hydroxyanisole. The LLOQ by Tanaka et al. was 0.10 µg/mL.

With the present method based on mass spectrometry we aimed to improve the literature method both for sample preparation and sensitivity. It is well known that SPME followed by gas chromatography coupled to mass spectrometry is a highly sensitive method and the electron ionization source (EI) owns a high fragmentation repeatability.

The sample preparation herein described is free from the necessity of derivatization to detect the redox reaction product 2-hydroxyanisole. The HS SPME with carboxen/polydimethylsiloxane fiber was improved by testing various pH buffers, catalyzer concentrations, reaction times and fiber exposure times, stirring of solution.

We found that the best conditions to obtain the highest amount of 2-hydroxyanisole were: Solution buffering at pH 2.5, 0.1 M catalyzer concentration, 1 h at 60 °C time reaction, time of 30 min of exposure of the fiber without stirring. In these conditions a LLOQ value of 0.05 µg/mL was obtained.

With this HS SPME-GC-MS method we measured the amount of H_2_O_2_ in fishery products, comparing fresh and illicit treated samples. In the latter case, the concentration should be hypothesized by extrapolating over the calibration curve range because the signal was too abundant, and it was calculated larger than 100 ppm, as listed in Table 4.

## 5. Conclusions

In conclusion, the developed mass spectrometry based analytical methods show to be suitable to notice the illicit treatment of fishery products with hydrogen peroxide. Both the UHPLC-MS method and the HS SPME-GC-MS method are applicable to detect molecules related to the use of hydrogen peroxide solution to whiten and refresh aged fish food.

By LC-MS, low molecular mass amines were detected with high selectivity and good sensitivity. The TMAO and TMA ratio was shown to be reversed by the illicit treatment, simulating an unreal apparent freshness of fishery products foods.

The use of hydrogen peroxide on fish products was confirmed by the measurement of 2-hydroxyanisole with HS SPME-GC-MS after a redox reaction between anisole and residual H_2_O_2_ in the extracted solution, by exploiting potassium ferricyanide catalysis. After the optimization of sample preparation for headspace solid-phase microextraction and redox reaction parameters, the method was suitable to quantify the H_2_O_2_ residues in fish food matrices. When fishery products, especially squid, were whitened with hydrogen peroxide, its amount was shown to be easily detectable.

## Figures and Tables

**Table 1:** MRM (multiple reaction monitoring) parameters and voltages for tandem mass analysis in a positive ion mode of dimethylamine (DMA), trimethylamine (TMA), trimethylamine-N-oxide (TMAO), and cadaverine (CAD). DP: Declustering potential; EP: Entrance potential; CE: Collision energy; CXP: Collision exit potential.

Compound	Q1 *m/z*	Q3 *m/z*	DP (V)	EP (V)	CE (V)	CXP (V)
**DMA**	46	30	96	10	37	13.5
**TMA**	60	44	90	10	24	11
**TMAO**	76	58	90	12	22	15
**CAD**	103	86	60	10	13	23

**Table 2:** Validation parameters for calibration curves in (I) pH 2.5, 0.1 M phosphate buffer, (II) Atlantic bonito extraction solution, (III) European squid extraction solution. Diff% slope: Difference % of the slope of the calibration curve; RSD% LLOQ%: Relative standard deviation % of accuracy of lower limit of quantitation; BIAS% of LLOQ: Inaccuracy of lower limit of quantitation; LLOQ: Lower limit of quantitation.

Compound	Parameter	Matrices		
		(I)	(II)	(III)
DMA	Selectivity %	1.80	2.50	3.42
	Diff% slope	4.74	6.53	7.84
	RSD% LLOQ	14.7	15.0	14.9
	BIAS% LLOQ	19.0	20.3	19.5
	LLOQ (ng/mL)	25.0	45.0	45.0
	Recovery %	95.2	87.6	85.1
TMA	Selectivity %	0.50	1.32	1.97
	Diff% slope	3.60	5.19	6.41
	RSD% LLOQ	2.16	7.86	4.36
	BIAS% LLOQ	20.0	19.8	19.0
	LLOQ (ng/mL)	30.0	70.0	70.0
	Recovery %	93.6	89.3	86.0
TMAO	Selectivity %	0.05	0.90	0.85
	Diff% slope	4.07	14.6	8.2
	RSD% LLOQ	8.60	10.6	9.63
	BIAS% LLOQ	9.50	12.7	18.6
	LLOQ (ng/mL)	30.0	50.0	50.0
	Recovery %	102.5	91.4	90.7
CAD	Selectivity %	0.03	0.40	0.60
	Diff% slope	6.85	8.97	12.3
	RSD% LLOQ	9.80	15.3	14.3
	BIAS% LLOQ	16.0	17.6	19.0
	LLOQ (ng/mL)	20.0	40.0	40.0
	Recovery %	99.8	94.1	85.8

**Table 3:** Concentration values of trimethylamine-N-oxide (TMAO) and trimethylamine (TMA) in real fish samples of Atlantic bonito (*Sarda sarda*) and European squid (*Loligo vulgaris*). The amount is expressed in mg/kg. Fresh referred to freshly caught samples; aged to samples left at room temperature for 4 h; H_2_O_2_ treatment to samples treated with hydrogen peroxide (see Material and Methods).

Sample	Compound	Fresh (mg/kg)	Aged (mg/kg)	H_2_O_2_ Treatment (mg/kg)
*Sarda sarda*	TMAO	1700 ± 238	170 ± 24	410 ± 57
	TMA	170 ± 24	1250 ± 350	720 ± 180
*Loligo vulgaris*	TMAO	1200 ± 336	140 ± 20	850 ± 212
	TMA	210 ± 29	970 ± 242	200 ± 28

**Table 4:** Quantitation of H_2_O_2_ in a 0.1 M phosphate buffer pH 2.5 solution and in fishery products samples. QC were quality control samples treated with hydrogen peroxide in controlled conditions (0.5 µg/mL of H_2_O_2_); five real samples were illegally treated with peroxide.

Matrix	Samples	H_2_O_2_ (µg/mL)
Buffer		0.5 ± 0.07
*Sarda sarda*	fresh	0.44 ± 0.11
*Loligo vulgaris*		0.43 ± 0.10
*Sarda sarda*	QC	>100
*Loligo vulgaris*		
*Sarda sarda*	real	>100
*Loligo vulgaris*		
